# “At home, no one knows”: A qualitative study of retention challenges among women living with HIV in Tanzania

**DOI:** 10.1371/journal.pone.0238232

**Published:** 2020-08-27

**Authors:** Godfrey A. Kisigo, James S. Ngocho, Brandon A. Knettel, Martha Oshosen, Blandina T. Mmbaga, Melissa H. Watt

**Affiliations:** 1 Kilimanjaro Clinical Research Institute, Moshi, Tanzania; 2 Duke Global Health Institute, Duke University, Durham, North Carolina, United States of America; 3 Kilimanjaro Christian Medical University College, Moshi, Tanzania; 4 The University of Cape Town, Cape Town, South Africa; 5 Kilimanjaro Christian Medical Centre, Moshi, Tanzania; Brown University, UNITED STATES

## Abstract

**Introduction:**

Despite the broad success of Prevention of Mother-to-Child Transmission of HIV (PMTCT) programs, HIV care engagement during the pregnancy and postpartum periods is suboptimal. This study explored the perspectives of women who experienced challenges engaging in PMTCT care, in order to better understand factors that contribute to poor retention and to identify opportunities to improve PMTCT services.

**Methods:**

We conducted in-depth interviews with 12 postpartum women to discuss their experiences with PMTCT care. We used data from a larger longitudinal cohort study conducted in five PMTCT clinics in Moshi, Tanzania to identify women with indicators of poor care engagement (i.e., medication non-adherence, inconsistent clinic attendance, or high viral load). Women who met one of these criteria were contacted by telephone and invited to complete an interview. Data were analyzed using applied thematic analysis.

**Results:**

We observed a common pathway that fear of stigma contributed to a lack of HIV disclosure and reduced social support for seeking HIV care. Women commonly distrusted the results of their initial HIV test and reported medication side effects after care initiation. Women also reported barriers in the health system, including difficult-to-navigate clinic transfer policies and a lack of privacy and confidentiality in service provision. When asked how care might be improved, women felt that improved counseling and follow-up, affirming patient-provider interactions, and peer treatment supporters would have a positive effect on care engagement.

**Conclusion:**

In order to improve the impact of PMTCT programs, there is a need to implement active tracking and follow-up of patients, targeting individuals with evidence of poor care engagement. Tailored supportive intervention approaches may help patients to cope with both the perceived and actual impacts of HIV stigma, including navigating disclosures to loved ones and accessing social support. Fostering HIV acceptance is likely to facilitate commitment to long-term treatment.

## Introduction

Early initiation of antiretroviral therapy (ART) during pregnancy, followed by lifelong adherence to treatment, has shown to be effective in preventing mother-to-child transmission of HIV (PMTCT) and improving maternal health outcomes [1, 2]. This clinical protocol, referred to as Option B+, was first recommended by the World Health Organization (WHO) in 2012 following successful implementation in Malawi [3]. Thereafter, Option B+ was adopted as policy by several countries in sub-Saharan Africa (SSA), including Tanzania, where PMTCT has been incorporated into routine antenatal clinic (ANC) services since 2015 and remains the standard of care. In the era of Option B+, Tanzania has successfully managed to increase identification of pregnant women living with HIV and link them to HIV services [4].

In eastern and southern Africa, where the majority of HIV cases are located, it is estimated that that 12 million women of reproductive age are living with HIV. Under Option B+ guidelines, all of these women should start or continue ART during their pregnancy, steps which have substantially reduced the rates of mother-to-child transmission, from 18% in 2010 to an estimated 9.9% in 2017. However, mother-to-child transmission remains responsible for 160,000 new infant infections each year, representing 9% of new HIV infections globally [5, 6]. Despite the successes of Option B+ in expanding PMTCT coverage, one-third of women who initiate or continue ART during pregnancy are lost to follow up (LTFU) six months later, which is substantially higher than LTFU in the general population of people living with HIV [7–10]. This high rate of LTFU has critical negative implications for the United Nations ‘90-90-90’ targets (i.e., by 2020, 90% of all people living with HIV (PLWH) will know their HIV status; 90% of all people with diagnosed HIV infection will receive sustained ART; and 90% of all people receiving ART will have viral suppression) [11]. The discontinuation of ART, as a result of LTFU, causes high viral load rebound that increases the chance of HIV transmission to the baby via breastfeeding, and to male partners via unprotected sex. Furthermore, inconsistent ART adherence may contribute to drug resistance, both in these women and in the broader population of people living with HIV [11].

Several studies have reported fear of unintended disclosure of one’s HIV status, stigma, lack of partner support, and structural barriers to be the leading causes of LTFU among people in HIV care [12–18]. Few of these studies, however, have elicited the perspectives of women who are LTFU or in care but experiencing retention challenges. The purpose of this study is to understand the factors that impede care engagement among pregnant and postpartum women living with HIV, and to identify opportunities to improve care engagement for this population.

## Methods

### Overview

This qualitative study was conducted as part of a longitudinal, mixed-method cohort study of 200 women who were enrolled in PMTCT care from clinics in the Kilimanjaro region of Tanzania [7]. We identified 42 women (21% of the cohort) who had indicators of poor care engagement during the study period and invited them to complete an in-depth interview. Twelve of these women agreed to complete an interview to discuss their experiences with HIV care during the pregnancy and postpartum periods, focusing on the factors that contributed to suboptimal care engagement. Data collection occurred between December 2017 and July 2018. The study received ethical approval from Duke University, Kilimanjaro Christian Medical Center, and the Tanzanian National Institute for Medical Research.

### Procedures

To identify potential participants with care engagement challenges from the broader cohort, we gathered data on self-reported medication adherence, completed a medical record review of clinic attendance, and gathered viral load data at 6 months postpartum. Poor care engagement was defined as meeting one or more of the following criteria: 1) self-report of stopping care or skipping medication for at least 30 days in a period of three months, 2) being identified as LTFU in the medical record, defined as missing three consecutive monthly HIV appointments without documentation of transfer, or 3) high viral load (≥1000 copies/ml) during a study-provided test at 6 months postpartum [7].

All women enrolled in the larger cohort study completed written informed consent and provided contact information upon enrollment, along with their preferences of how they could be reached for future follow-up. We attempted to contact all 42 eligible women by telephone, home visits, or in-person during a study visit. Twelve (29%) agreed to participate. Enrolled women had sought care and been enrolled in the cohort study at five different clinics in the region. Among the 12 women, 5 had a self-report of an extended period of missing ART medication, 4 were identified as LTFU in the medical record, and 3 had a high viral load. Among 30 women who met criteria for poor care engagement but were not interviewed, 22 were unreachable, 3 declined to schedule an interview, and 5 had moved away from the Kilimanjaro region.

Participants agreeing to take part in an in-depth interview were invited to schedule a time and choose their preferred location of interview (e.g., the clinic of enrollment, another clinic, home). The interviews were conducted in Swahili by a trained Tanzanian researcher who had prior experience with qualitative research and had no prior affiliation with the study clinics. Interviews took approximately one hour and were audio-recorded with the participant’s consent. Following the completion of the interview, the audio files were transcribed and translated into English by a research assistant fluent in English and Swahili.

### Data collection instruments

The development of the semi-structured interview guide was informed by the preliminary findings of the larger cohort study [7]. The guide included open-ended questions and probes related to care engagement and adherence (including both challenges and facilitators of care engagement), choice of clinic and mobility, and plans for HIV care in the future (see Table 1 for additional detail). Each section of the guide began with an opening question, followed by potential probes to be used to explore the topic in greater depth.

**Table 1:** The content of the in-depth interview guide.

Care engagement and adherence
Experience getting diagnosed
Reaction to diagnosis
Disclosure and social support
Giving birth and being a new mother
HIV treatment experience
Choice of clinic and mobility
Location of the first and current clinic
Experience/retesting at another clinic
Reasons for choosing/switching clinic
Experience of moving or traveling out of the clinic area
Long-term treatment plans
Knowledge of ART treatment duration
Feeling about taking ART for life
Transfer to CTC
Sense of the future
Vision about the future as a mother
Impact of HIV
Plans for the child
Reflection on discussion
Experience speaking about HIV
Willingness to talk about HIV in the future

### Analysis

Data were analyzed using applied thematic analysis [19] with an emphasis on qualitative memo writing [20]. The applied thematic approach is a rigorous set of inductive procedures designed to identify and examine themes from textual data in a way that is transparent and credible. After multiple readings of the transcripts, document memos were written for each individual interview. Memos followed an established template of *a priori* domains, informed by the interview guide, to extract and synthesize the core meaning from text related to the research questions and to pull out representative quotes. Memos were written by 2 individuals [GAK, BAK]; each transcript/memo pair was developed by one analyst, and then reviewed, discussed, and revised in consensus building meetings with the other analyst to confirm the completeness and rigor of the memos. On average, each full transcript represented 20 pages of single-spaced text and was condensed to 5 pages of text in the memo writing process. The memo writing process identified emerging themes and informed the development of a structured codebook focused on care engagement challenges and opportunities to strengthen care engagement. The codebook included six components: the code, a brief definition, a full definition, guidelines for when to use the code, guidelines for when not to use the code, and examples. The 12 document memos were uploaded to NVivo software and coded using the final codebook. After coding, code-level queries were run, and analytic memos were written to synthesize the content, make comparisons across participant characteristics, and draw deeper meaning on each theme. The original transcripts were re-visited as needed throughout the coding process to ensure emerging themes were represented and to further contextualize the findings.

## Results

### Participant demographics

Among the 12 women interviewed in this study, the average age was 27 years (range 19–37). Six women were in a relationship but not married, five were married, and one was single. Seven women had a known HIV diagnosis before the index pregnancy, while five were newly diagnosed during the index pregnancy. Four women were pregnant for the first time. Eight women had disclosed their HIV status to the father of the baby. All but one woman was employed in a formal sector. Additional, individual-level details of the 12 study participants are presented in Table 2.

**Table 2:** Description of the sample of women living with HIV and experiencing care engagement challenges (n = 12).

ID	Age	Relationship	Highest Education	Employed?	Disclosed to anyone	Disclosed to partner	Parity	Time since HIV diagnosis	Treatment adherence
01	32	In relationship	Primary	No	Yes	Yes	2	< 1 year	In care, missing pills
02	23	In relationship	Secondary	No	No	No	0	< 1 year	Out of care
03	20	In relationship	Primary	Yes	Yes	Yes	0	< 1 year	Out of care
04	37	Married	Primary	No	Yes	Yes	4	8 years	In care, high viral load
05	28	Married	Primary	No	Yes	Yes	1	< 1 year	Out of care
06	28	In relationship	Primary	No	Yes	Yes	2	6 years	In care, high viral load
07	26	In relationship	No education	No	Yes	No	3	4 years	In care, missing pills
08	19	Single	Secondary	No	Yes	No	0	8 years	Out of care
09	32	Married	Primary	No	Yes	Yes	3	3 years	In care, missing pills
10	22	Married	Primary	No	Yes	Yes	1	< 1 year	In care, high viral load
11	37	Married	Primary	No	Yes	Yes	3	3 years	In care, missing pills
12	26	In relationship	Primary	No	Yes	No	0	1 year	In care, missing pills

The qualitative findings are presented based on the two research objectives that guided the analysis: 1) retention in care challenges and 2) opportunities to strengthen care engagement.

### Retention in care challenges

Women reported facing multiple and complex challenges that undermined their efforts to remain actively engaged in PMTCT care during the pregnancy and/or postpartum periods. These challenges included barriers at various levels (i.e., intrapersonal, interpersonal, and structural). The emerging themes below are ordered by their saliency in the analysis.

#### Fear of stigma

Participants reported a wide range of stigma experiences, most notably being the subject of gossip, isolation, and social rejection. In addition, they feared or anticipated these stigmatizing reactions if their HIV status were to become known by others. These experiences and anticipations were described as key barriers to HIV care engagement in multiple ways. First, the blame and judgment that women experienced led them to lose hope for their future life, and therefore to lose motivation to engage in care.

“I went through a lot of challenges, and I was discouraged (to continue HIV care). Like the way… relatives isolate you, talk bad things about you, and when you come to the other side of the family, his (partner’s) relatives want to blame you, and when it comes to a problem (HIV infection), they can’t even support you.”.

(PID 05; Out of care)

Second, after being stigmatized, women were subjected to a heightened feeling of being unwanted, which led to poor or discontinued care engagement.

"When I went home, my mother had stigmatized me because she knew about my HIV infection. I felt that I should isolate myself and stay alone so that I won’t feel depressed or feel bad about my (HIV positive) status."

(PID 07; In care, missing pills)

Lastly, women feared that people would learn about their HIV status and, thereafter, stigmatizing them. This fear led women to avoid their clinic appointments, as they did not want to be seen at the PMTCT clinic and labeled as someone living with HIV.

“When someone sees you every month at the clinic, they might ask, ‘Why is she coming every month?’ You know, the clinic is near the place I am staying, and many people know me. So they will ask themselves, ‘What is wrong with this person every time she is going to the hospital?’”

(PID 10; In care, high viral load)

Among women who were newly diagnosed during index pregnancy, the fear of stigma and unintended disclosure was amplified during the postpartum period. Pregnancy care normalized and justified women’s regular clinic attendance, but in the postpartum period women were newly confronted with the fears of involuntary HIV disclosure.

“The reason which made me to stop going to the clinic for refill is the change in my situation. Before I was pregnant, and now I have a baby. When I am taking my baby to the clinic, all the procedures and steps are known, and therefore, I cannot use that as an excuse to attend my clinic.”

(PID 02; Out of care)

#### Lack of HIV disclosure

All but one participant had disclosed her serostatus to at least one individual, but disclosures were highly selective, were based on the strength of the relationship, and were reliant on perceived trust. Participants described feeling more comfortable disclosing to a partner or close family member, while disclosures to friends and neighbors were rarer. Nearly all disclosures were closely linked to perceptions of whether the person would respond in a supportive manner, and avoidance of disclosure was common when participants anticipated a stigmatizing response. About one-third of participants had concealed their HIV status to the partner or father of the baby. These women reported living under constant stress associated with fear that a partner might discover their HIV positive status if they continue to use ART in secrecy.

“When I went there (partner’s house), the biggest challenge that I got was to use the medicine. I had to swallow them secretly (because she did not disclose her HIV status to the partner)”

(PID 12; In care, missing pills)

Likewise, women who had hidden their HIV status from immediate family members who lived in the same home expressed similar anxieties about involuntary disclosure.

“It was tough, and still it’s tough for me in my life. The surroundings I am living in …at home, no one knows, so that’s the way I am doing it (taking medication) in secret.”

(PID 02; Out of care)

Among women who chose to disclose their status, several reported experiences of discrimination and stigma.

“I disclosed it on the same day I was diagnosed. I told him (my partner) the test results are bad (HIV positive). He said okay; he took all of his clothes and moved to the next room. From there onwards there was no communication.”

(PID 04; In care, high viral load)

“I was planning to disclose to two people at first. I knew that they would give me hope, but after the disclosure, they panicked and we had to do a family meeting.”

(PID 05; Out of care)

In the process of seeking HIV services, the majority of the women expressed concerns about the clinic set-up and the flow of services, which they felt compromised patient privacy and confidentiality. Some of the antenatal clinics had a separate room for PMTCT services. Therefore, pregnant and postpartum women living with HIV were attended separately, but other patients were able to discern their HIV status based on the room they entered for the appointment. Moreover, participants were concerned that healthcare providers might disclose their status to others.

“So where is confidentiality? I am there and she (the nurse) asks me what have I come here for (in front of other patients). Will I mention that I am here to take my (HIV) medications?”

(PID 10; In care, high viral load)

#### Lack of social support

Many women noted challenges related to social support, particularly from the partner or father of the baby. The majority of these women had financial constraints prior to the pregnancy and HIV diagnosis. This lack of financial support led women to endure episodes of hardships, including lack of transport fares to attend the clinic or insufficient food, which hampered adherence to medication.

“Honestly speaking, after I gave birth, getting care was a problem to me. I remember even the bus fare from home to the clinic was a problem. When I tried to contact the father of my child [for support], he said I should leave him alone.”

(PID 08; Out of care)

Of note, one woman reported experiencing intimate partner violence after being diagnosed with HIV. The woman explained that her partner was also living with HIV, but he had refused to seek care.

“When I started HIV treatment, my partner was obsessed with alcohol. He would return home from work drunk with no money. He would insult and beat me. He ripped up the two clinic cards [used for tracking appointments and drug refills at the clinic] and threw away my pills. I could not tolerate that and keep up with HIV care, so I decided to quit.”

(PID 05; Out of care)

#### ART side effects

All women reported experiencing moderate to severe short-term side effects of ART, including dizziness, nightmares, and tiredness. To some women, these symptoms had impacted their quality of life to the extent of hindering their ability to engage in routine activities and to maintain good adherence.

“It seems the medication is too strong because the problem (dizziness and heavy sleep) is not mine alone. Everyone who is using it (ART) experiences the same problem.”

(PID 09; In care, missing pills)

“I mean, they (ART) made me feel dizzy, and I almost fell from the chair. Because of this feeling, there was a time I stopped taking my medicine.”

(PID 12; In care, missing pills)

#### Distrust of the HIV diagnosis

Four women reported having difficulty accepting the validity of their HIV test results, which led to delays or avoidance of initiating HIV care. Among these women, some never initiated care, while some sought to re-test at two or more different clinics prior to initiating care for the sake of protecting the unborn baby, but they still held doubts about their HIV status that would influence later decisions to drop out of care.

“At first, I didn’t believe it. The next day I went to another facility and they also tested me and found me with illness (HIV infection). Then my husband said, ‘If you don’t believe it, let me take you to a different clinic.’ We went there and the test result was the same.”

(PID 10; In care, high viral load)

Among women who distrusted the HIV diagnosis, but nevertheless initiated care, their motivation for care often centered on the health of the unborn child. Therefore, HIV care engagement was not considered essential for their well-being beyond pregnancy and the early post-partum period. For example, one woman who lost her baby said:

"You know, I did not receive the test results well. But I said to myself, because I am pregnant, let me do it (initiate care). I will use these medications for a year so that my baby would not get any problem (HIV infection). However, I planned to quit the medication after that year because I do not need them."

(PID 03; Out of care)

#### Transfer system

Women noted several barriers to accessing and utilizing PMTCT services. Among the barriers reported, the referral mechanism was a key challenge. In the study clinics, once a woman was diagnosed with HIV in one clinic, she was required to present a formal referral letter from that clinic to seek care elsewhere. Some women who had emergency travels or an extended stay out of town were unable to get refills of their medication in other clinics without an official referral letter, even though they had presented their clinic card.

“I went to a nearby clinic. I had no referral letter, so they refused to prescribe my medication, although I was given a few of them for the child.”

(PID 07; In care, missing pills)

### Opportunities to strengthen care engagement

The central theme that emerged in discussions about opportunities to enhance care engagement was women’s desire for improved social support. Consistently, women asserted that their HIV diagnosis and pregnancy had brought new challenges requiring additional, continuous support from partners, family members, social networks, and health care providers. The following sub-themes outline the support needed, as narrated by women in this study.

#### Individually tailored counseling and follow-up

Women reported receiving counseling about PMTCT on the day of receiving their HIV diagnosis. Women with an established diagnosis described receiving counseling from the nurse in antenatal care. Participants described the counseling as mostly focused on the interests of the unborn baby. Thus, the goal was to ensure pregnant women initiate HIV care early in the antenatal period, restart, or continue taking ART to reduce the likelihood of mother-to-child transmission.

“At the pregnancy clinic, she (the nurse) told me that if you adhere to your medications, the baby will be born well, and if you do not adhere, the baby will come out with the infection, and I started to reflect on that.”

(PID 04; In care, high viral load)

Participants reported receiving little or no counseling in subsequent visits to address emerging challenges in the continuum of care (e.g., how to manage disclosure or stigma). Women articulated there was an unmet need for sustained and tailored counseling, especially for women with signs of poor care engagement.

“The most important thing for them (women with poor care engagement) is to be counseled about good adherence, and the provider (nurse or doctor) needs to understand the problems they have.”

(PID 08; Out of care)

Other women felt that home visits for women with poor care engagement might encourage women to describe the challenges they are experiencing and return to the clinic.

“They (health care providers) should visit the patient if she does not attend the clinic for a long time, so they know why is she not coming to take her medication, and what is the problem.”

(PID 05; Out of care)

#### Affirming patient-provider interactions

Women acknowledged being motivated to remain engaged in care when nurses and other healthcare workers provided emotional support and encouragement during follow-up visits at the clinic. Also, women asserted that affirming health care providers and clear communication from healthcare workers improved their quality of care. When women felt more personally connected to healthcare workers, they described feeling more confident that they would not be stigmatized in the health system and that their privacy would be respected.

“Nurses are generous, they care about the patient, they are close with the patients to the extent where you can call the nurse if you have a problem, and she can help you. So, I feel that I receive excellent care”

(PID 09; In care, missing pills)

#### Treatment supporters

When asked whether they would like to receive additional support in their care from a clinic-based treatment supporter, all but one woman desired to have this service. Also, some women felt they could benefit more from peer treatment supporters (i.e., someone living with HIV), as that person would be more likely to understand their situation and unique challenges. Peer support programs were not asked about specifically in the interview guide, but the topic did emerge naturally in several of the interviews. None of the clinics were offering peer support programs at the time of the interviews, but programs have since been implemented at several clinic sites.

“I see it (peer support) as a good thing because the other patients I talk to, when I ask them, are you going inside that room (PMTCT clinic)? They reply yes, and we start to advise each other. I feel like it comforts me. Even if I am stressed, I feel good.”

(PID 04; In care, high viral load)

## Discussion

**Fig 1:**
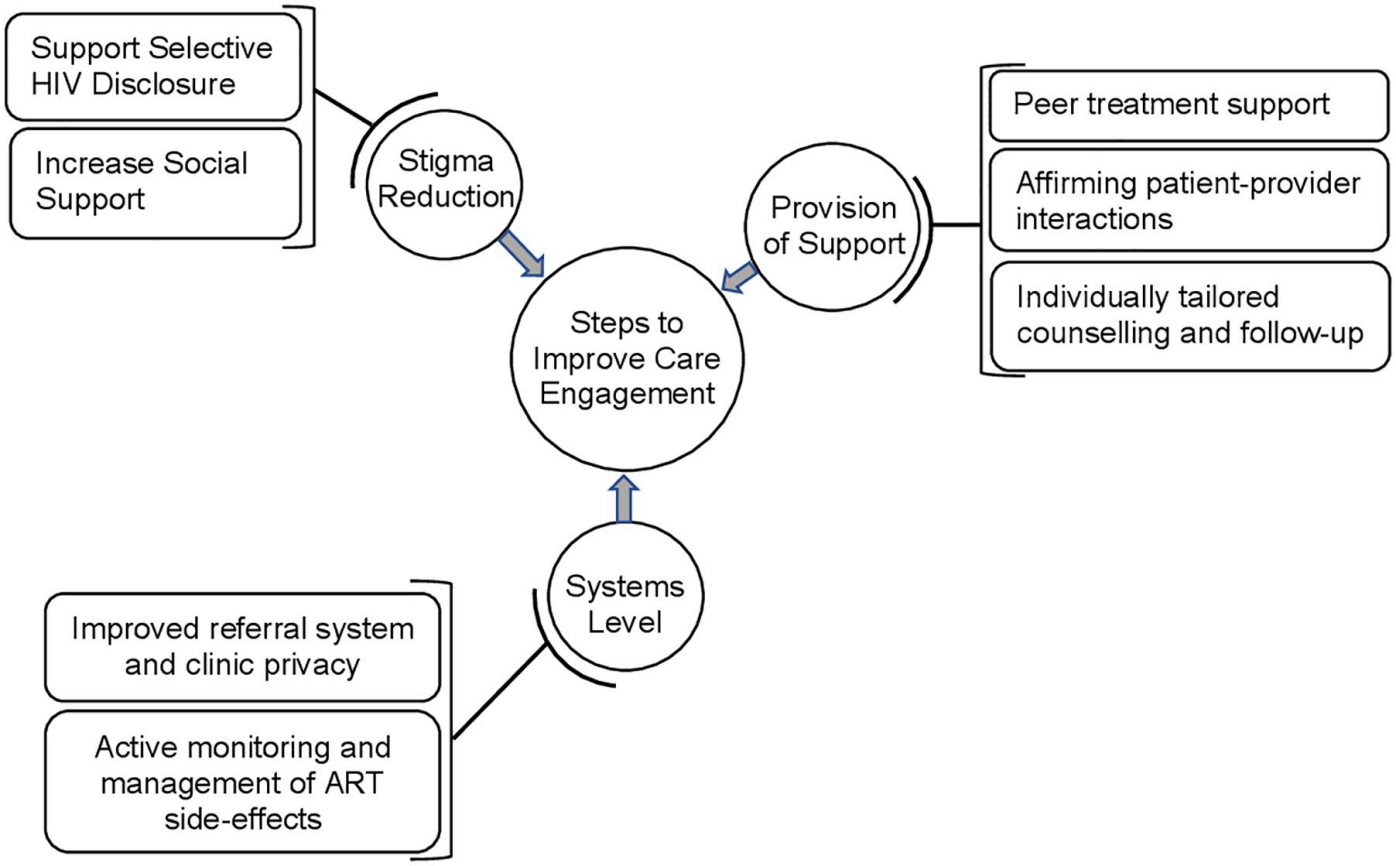
A framework of interaction between challenges, opportunities, and care engagement as reported by women.

This study explored challenges to HIV care engagement and retention in care, and also identified opportunities to support care engagement in PMTCT services during both the pregnancy and postpartum periods. We found that enacted or anticipated stigma contributed to a lack of HIV disclosure and reduced opportunities for social support. “Women commonly distrusted the results of the HIV test, leading to avoidance of initiating HIV care. Several women who initiated ART reporting experiencing medication side effects, which contributed to early discontinuation of care. Participants also described several challenges in the health system, including difficult-to-navigate clinic transfer policies and a lack of privacy and confidentiality in service provision, which had a negative impact on care engagement. When asked how care might be improved, women felt that improved counseling and follow-up, affirming patient-provider interactions, and peer treatment supporters would have a positive effect on HIV care engagement (Fig 1). These findings contribute to prior studies highlighting the multiple, complex barriers that can impede care engagement among pregnant and postpartum women, including individual, social, and health systems factors [6, 8, 22]. A variety of interventions have demonstrated preliminary effectiveness in addressing these challenges and improving care retention, including task-shifted support from community health workers, engaging male partners in HIV testing and counseling, provision of ART in community-based adherence clubs, and mobile reminders for medication adherence and clinic appointments [23–25]. While our findings echo many of the care engagement challenges faced in the general population of PLWH, women who initiate HIV care during pregnancy face unique challenges as they traverse the antenatal continuum. Our findings highlight the specific challenges as women transition to postpartum care, and the need for targeted support during this period.

The shifts toward universal HIV testing in antenatal care and immediate initiation on treatment for those who test positive, as recommended in the Option B+ policy, have significantly decreased rates of mother-to-child transmission of HIV [1, 2]. This success has been facilitated by increases in the identification and enrollment of HIV positive pregnant women into HIV care [15, 21]. However, the universal nature of testing during pregnancy may mean that some women are not prepared to test, to accept a positive test result, or to immediately initiate HIV care [22]. The shock of a new diagnosis may also contribute to feelings of doubt about a positive test result and lack of acceptance.

The postpartum period is also a critical stage for HIV care engagement, not only to prevent HIV transmission via breastfeeding but also to prepare women to transition into long-term care and treatment for HIV beyond PMTCT. Thus, at the beginning of HIV care, it is essential to deploy strategies to foster acceptance of an HIV diagnosis, ensure adequate social support is in place, and build women’s intrinsic and long-term motivation to remain in HIV care [7]. These supportive counseling interventions should be repeated during the postpartum period, as women may face additional challenges with motivation, anticipated stigma and disclosure. Pregnant women commonly cite the desire to avoid mother-to-child transmission as a primary driver of care engagement; thus, they may be prone to dropping out of care after the birth of the child. These types of care challenges may be even more pronounced among women who experience a major life transition during pregnancy or the postpartum period, such as a miscarriage, stillbirth, or death of a young child [23].

Stigma is a prominent barrier to the success of HIV prevention and treatment programs in the general population. Likewise, women enrolled in PMTCT services are negatively impacted by stigma at each step of the care continuum [12, 21, 24–28]. Furthermore, the pregnancy adds another layer of vulnerability, especially for women who rely on their partners for financial support. This may be particularly true in settings where gender inequity limits opportunities for formal employment among women. Under these circumstances, women may choose not to disclose their HIV status to the partner and immediate family members because of fear of social exclusion and loss of support [7, 18]. To assist women to navigate this terrain, PMTCT services should incorporate tailored counseling interventions that focus on empowering and supporting women in their decision-making related to HIV disclosure (i.e., choosing HIV disclosures with fewer possibilities of negative responses), in order to help them mitigate individual and community stigma. Also, based on evidence from previous studies [29–32], male engagement in PMTCT services has a potential to strengthen HIV care engagement among women. Since Tanzania is already implementing HIV couple testing and counseling during the first ANC visit [33], further studies are recommended to examine available opportunities to enhance male involvement in PMTCT services, including provision of treatment support, beyond the first ANC visit.

Our data suggest that health care providers and clinic-based treatment supporters have an essential role in strengthening HIV care engagement during the pregnancy and postpartum periods. Literature in patient-centered care has shown that affirming patient-provider interactions (e.g., use of non-judgemental communication) has the potential to enhance positive experiences in seeking health care, which was also reported by women in our study [34, 35]. Although Tanzania faces a critical shortage of health care providers, it is difficult for healthcare workers to spend sufficient time with each patient, to identify patients with poor care engagement and address their challenges at the individual level. Also, the use of peer treatment supporters can be used in clinics to ‘task-shift’ aspects of adherence counseling and improve PMTCT care engagement, particularly in settings where a shortage of health workforce is prominent [36, 37]. Furthermore, the use of community health workers (CHWs) holds a greater promise in improving the quality of care by complementing the standard of care at the community level [30, 38, 39]. The involvement of CHWs in PMTCT services would further reinforce the capacity of health facilities to actively follow up and tracking women who have signs of poor engagement or who are lost to follow-up.

The strength of our study is that we interviewed women who were recently LTFU or were in care but experiencing retention challenges. Thus, the findings are drawn from a sample of women most impacted by the gaps in HIV care. Also, women reflected on the real-time experiences of HIV care engagement, which minimized the risk of recalling bias. However, we recognize the following limitations. First, we were unable to recruit the majority of women who met the criteria of poor care engagement, which was an expected challenge given many of these women were already out of care. However, women who were unreachable or unwilling to participate may have had more intense or different challenges in their care retention as compared to the women who agreed to be interviewed. Second, although participants were assured that their information would be kept confidential, women might have under-reported challenges related to service provision due to fear that the research team might share information with health care workers. Lastly, given the challenges of recruiting women who have disengaged from care and resulting small sample size, we could not perform further analysis to explore the scope of care engagement challenges by sub-group of women (e.g., new vs. established diagnosis, single vs. multiple parity). Future studies may seek to recruit larger samples of women disengaged from care to perform sub-analyses based on individual and social factors.

## Conclusion

Consistent HIV care engagement during the pregnancy and postpartum periods is critical for the success of PMTCT programs. To improve retention in care, there is a need to identify individuals with evidence of poor care engagement, via active surveillance of clinic attendance records and viral load test results. Tailored intervention approaches are needed to help these patients to cope with both the perceived and actual impacts of HIV stigma, including navigating HIV disclosure and improving access to social support. Fostering HIV acceptance may facilitate commitment to long-term treatment that starts during pregnancy and extends throughout women’s lifetimes.

## Supporting information

S1 Appendix (DOCX)
