# Supplementary material for: “At home, no one knows”: A qualitative study of retention challenges among women living with HIV in Tanzania
Source: PLoS One. 2020 Aug 27;15(8):e0238232. doi: 10.1371/journal.pone.0238232 (PMC7451655; doi:10.1371/journal.pone.0238232)
Supplement: S1 Appendix — (DOCX) [file pone.0238232.s001.docx]

**IN-DEPTH INTERVIEW GUIDE**

Thank you for agreeing to spend some time talking with me today. Our conversation will take about an hour, and the tape recorder will be used to make sure that nothing will be missed during our interview. What you say to me today will be confidential, and I will not share any information with your health care providers. The purpose of these interviews is to understand the experiences of women with HIV, so that we can support them better. I hope you will feel comfortable to speak freely and honestly with me. Do you have any questions or concerns before we begin?

1. **Introduction**

I know that you’ve had a chance to talk to other members of our research team (like Mama Pilli) over the last year. I’d like to start by getting to know you better. Can you tell me a little about yourself.

[NOTE: Give her the opportunity to talk briefly about herself. Let her know that you’ll come back to some of these issues later in the interview.]

1. **Care engagement**

Let’s talk about how you’ve been doing with your HIV care and taking your medication. As a reminder, I want you to feel comfortable and honest talking to me about this – you don’t need to feel shy or embarrassed about any difficulties you’ve had.

Can you begin by telling me what it’s been like for you since you first learned that you have HIV?

*Try to follow her story/direction, because we really want to see what comes up as most important to her. Some possible probes or ways to get conversation flowing:*

- ***Day of diagnosis***: how she felt learning about her diagnosis and being told that she would take ARVs for the rest of her life; any doubt she had about her diagnosis; counseling she received (or didn’t); what would have been helpful at that point
- ***Experience/feelings about the pills***: how/whether she believes they work, worries/experiences with side effects
- ***Relationship with partner***: disclosure, trust, support
- ***Relationship with others***: disclosure, trust, support
- ***Being pregnant***: what was it like for her during pregnancy, how she felt being HIV+ and pregnant, how/when she took her pills (and when/why she didn’t)
- ***Giving birth***: what was it like for her giving birth, how she felt being giving birth as a woman with HIV, how/when she took her pills during labor/delivery (and when/why she didn’t)
- ***Being a new mom****:* what was it like for being a new mom, how she felt being giving birth as a woman with HIV, how/when she took her pills after birth (and when/why she didn’t)
- ***Current care engagement****:* Confirm whether she is currently attending the clinic and taking her medication (if not, probe deeply on what led her to stop)

1. **Choice of clinic, mobility**

I’m interested to hear more about *where* you got your care, both care for your HIV and any other care for yourself or your baby.

*Possible probes (and confirmation):*

- Where did she *first* learn about her HIV diagnosis
- Did she go to any other clinic at that time (e.g., switching sites or getting confirmatory test) *If yes, understand reasons and experience
- Why she chose the clinic where she got her PMTCT care
- What was good / bad about pregnancy care in that clinic
- Did she ever want to be at a different clinic for her care
- Any experience with other clinics during her pregnancy
- Where she gave birth and why
- Where she got her care after giving birth and why
- What was good / bad about postpartum (+ baby) care in that clinic
- Any experience moving or being out of the area
- **If she ever changed clinics, how did she make the *transfer*

1. **HIV care engagement and adherence**

I really appreciate your taking the time to tell me more about your life and to share with me some of your challenges attending the clinic and taking medication. From what you have told me, you have faced the following challenges taking your medication: [SUMMARIZE WHAT SHE SAID]. Does that seem accurate? What else would you add to that?

Can you tell me about any other times that you have missed clinic appointments or not taken your pills? [ALWAYS PROBE FOR MORE TIMES]

Sometimes there are other challenges that exist, even if they don’t lead to us missing our pills. Can you tell me about anything else that we haven’t talked about yet that you find difficult in attending appointments or taking your medications.

*Possible probes, if these issues haven’t come up:*

- Challenges in reaching the clinic
- Financial issues that affect care
- Worries about being seen at the clinic (e.g., stigma, discrimination)
- Hiding medication
- Forgetting
- Worries
- Side effects
- Mistrusting/doubting diagnosis
- Believing she was cured
- Mistrusting ARVs
- Believing in prayers
- Do you know person/people who were in care and they are no longer taking their medications? What do you think could be the reason?

Tell me the things that have helped you to take your medication – like the strategies you use to take them.

1. **Long-Term Treatment Plans**

What have you been told about how long you will take ARVs?

- Who told her and how
- Questions she still has about this

How do you feel about taking medication for the rest of your life?

- Her own plans for whether she will continue taking ARVs
  - Motivations
  - Things that will be difficult (e.g., time, disclosure, travel)
  - Things that worry her (e.g., side effects)
- Where she’ll get care once she is done at the mother-baby clinic
  - What she thinks that will be like

1. **Sense of the Future**

I’d like to hear your thoughts for the future. What plans do you have now?

When you think of your future, what you are excited about, and what you are worried about?

- How she sees her future as a mom
- What are her future plans as a woman
- What she’d like for her child
- How HIV impacts how she sees her future
- Any plans to travel/move
  - Why? What is driving this?
- Hopes or plans for the baby – what are you excited about? What are you worried about?

1. **Conclusion**

What are your thoughts about how we can support women like you to stay in HIV care after they have had a baby?

How did it feel for you to speak to me, now after you’ve had your baby?

- Anything helpful about the conversation
- Whether this type of conversation might help other women

Do you have anything else you want to add before we end?

Thank you for taking the time to talk with me. Your views and experiences will help us to provide support for postpartum women in this community.

**Muongozo wa Mahojiano ya Kina**

Asante kwa kukubali kutumia muda wako kuzungumza na mimi leo. Mazungumzo yetu yatadumu kwa takribani saa moja, na tutatumia kinasa sauti kuhakikisha hatuachi kitu kutoka kwenye mahojiano yetu. Chochote utakachoniambia kitabaki kuwa siri, na sitatoa taarifa yako yoyote kwa muuguzi wa kituo chako cha tiba. Lengo la mahojiano haya ni kuelewa uzoefu wa wanawake wenye maambukizi ya VVU, ili kusudi tuwezi kuwasaidia vizuri Zaidi. Natumaini utajisikia vizuri kuongea na mimi kwa uhuru na ukweli. Je una maswali au shida yoyote ungependa kuuliza au kunishirikisha kabla hatujaanza mazungumzo yetu?

1. **Utangulizi**

Nafahamu ulipata nafasi ya kuzungumza na watafiti wengine wa mradi huu (Kama Sr. Mariki) mwaka uliopita. Ningependa kuanza kwa kukufahamu wewe vizuri. Je unaweza kuniambia kwa kifupi kuhusu wewe.

[ZINGATIA: Mpe nafasi ya kuongea kuhusu yeye mwenyewe kwa kifupi. Mjulishe kuwa baadae utaweza kurejea na kuuliza maswali juu ya utambulisho wake.]

1. **Ushiriki kwenye Huduma**

Sasa tuongelee hali ilivyo ya kuendelea kupata huduma yako ya VVU na kumeza dawa za ART. Kwa ajili ya kukumbusha, ninapenda ujisikie kuwa huru na muwazi katika kuongea na mimi kuhusu swala hili – Huhitaji kuona aibu au kujisikia vibaya kwa ugumu wowote uliokutana nao.

Unaweza kuanza kwa kuniambia hali yako ilivyo tangu ulipogundua una maambukizi ya VVU?

*Jaribu kusikiliza historia yako kwa ukaribu:*

- ***Siku ya utambuzi wa ugonnjwa***: Jinsi alivyojisikia alipogundua ana maambukizi na kuambiwa atatumia dawa za ARVs kwa maisha yake yote; mashaka yoyote aliyokuwa nayo kuhusiana na utambuzi wa ugonjwa; ushauri aliopata (au kutopata); kipi ambacho kingekuwa cha msaada kwa wakati huo.
- ***Uzoefu/Hisia kuhusiana na Vidonge:*** Kwa namna gani/je anaamini zinasaidia, hofu/uzoefu na athari za dawa zisizotarijiwa
- ***Mahusiano na mwenza***: Uwazi, kuaminiana, kusaidiana
- ***Mahusiano na wengine***: Uwazi, kuwaamini, msaadawao
- ***Ujauzito***: Hali ilikuwaje wakati wa ujauzito, Alijisikiaje kuwa mjamzito mwenye maambukizi ya VVU, namna gani na wakati upi alimeza vidonge vyake (na wakati upi na kwanini hakumeza)
- ***Kujifungua:*** Hali ilikuwaje wakati wa kujifungua, alijisikiaje kujifungua hali akiwa na maabukizi ya VVU, kwa namna gani/wakati upi alimeza vidonge wakati wa uchungu/kujifungua (na wakati upi/kwaninihakumeza)
- ***Kuwa mzazi****:* Anajisikiaje kuwa mama, kwa namna gani/wakati upi alimeza vidonge baada ya kujifungua (na wakati upi/kwaninihakumeza)
- ***Ushiriki wa huduma kwa sasa:*** Hakikisha kama kwasasa anahudhuria kiliniki na kumeza dawa zake (Kama hapana, dodosa kwa kina sababu uliyompelekea kusitisha huduma)

1. **Uchaguzi wa kliniki, Uhamaji**

Ninashauku ya kusikia zaidi kuhusu mahali unapopatia huduma, huduma ya VVU na huduma nyingine yeyote ya afya yako au mtoto.

*Dodosa (na hakikisha):*

- Mahali pa kwanza alipogundulika kuwa na VVU
- Je alienda kliniki nyingine kwa wakati ule (mfano., kubadili kituo au kufanya kipimo cha kuhakiki) *kama ndiyo, ulizia sababu na uzofeu wa kufanya hivyo
- Kanini aliichagua kliniki aliyopatia huduma ya PMTCT
- Kipi kilikuwa kizuri/kibaya kuhusiana nahuduma ya ujauzito katika kliniki hiyo
- Je aliwahi kutaka kuwa kwenye kliniki nyingine kwa ajili ya huduma yake
- Uzoefu wowote kuhusiana na kliniki zingine wakati wa ujauzito
- Alijifungulia wapi na kwanini
- Alipatia wapi huduma baada ya kujifungua na kwanini
- Kipi kilikuwa kizuri/kibaya kipindi kwenye huduma baada ya kujifungua (+ mtoto) katika kliniki hiyo
- Uzoefu wowote wa kuhama au kuwa mbali na eneo/mji ilipo kliniki
- **Kama amewahi kubadili kliniki, alifanyaje uhamisho

1. **Ushiriki katika Huduma ya VVU na Uzingatiaji**

Kuna wakati kunakuwa na changamoto, japo hazipelekei kufanya ukose kumeza dawa zako. Unaweza kuniambia jambo lingine lolote ambalo hatujaliongelea na unaona linakupa ugumu kufika kliniki au kumeza dawa zako.

*Dodosa mambo haya kama hayakujitokeza kwenye mazungumzo:*

- Changamoto katika kuja kliniki
- Maswala ya kifedha yanyoathiri huduma
- Hofu ya kuonwa na watu kliniki (km., Unyanyapaa, kutengwa) (km., stigma, discrimination)
- Kuficha dawa
- Kusahau
- Hofu
- Madhara ya dawa
- Kutoamini/mashaka na utambuzi wa ugoinjwa
- Kuamini amepona
- Kutokuamini ARVs
- Kuaminini katika maombezi na sala
- Je unamfahamu mtu/watu ambao walikuwa wanapata huduma za VVU na sasa wameacha kutumia dawa? Je unafikiri nini inaweza kuwa sababu?

Niambie vitu vilivyokusaidia kumeza dawa zako – kama mbinu unazotumia kuhakikisha unameza dawa.

1. **Mipango ya muda mrefu ya matibabu**

Je umeambiwa ni kwa muda gani utapaswa kutumia dawa za ARVs?

- Ni nani aliyemwambia na kwa jinsi gani
- Maswali aliyonayo kuhusiana na swala hili

Je unajisikia kuhusu kumeza dawa kwa muda wa maisha yako yote?

- Mipango yake kama ataendelea kutumia ARVs
  - Msukumo
  - Vitu vitakavyokuwa vigumu
  - Vitu vinavyompa hofu
- Utapatia wapi huduma baada ya kumaliza huduma katika kliniki ya mama na mtoto.
  - Anafikiria hali itakuwaje huko kwengine

1. **Mtizamo wa baadae**

Ningependa kusikia mawazo yako kuhusiana na baadae/yajayo. Una mipango gani kwasasa?

Ukifikiria kuhusiana na baade, kitu vitu gani vinakufanye uwe na shauku, na vitu gani vinakupa hofu?

- Anaionaje baadae kama mama
- Nini mipango yake ya baadae kama mama
- Kipi angependa kwa ajili ya mwanae
- Jinsi gani VVU inaathrir mtizamo wake wa baadae
- Mipango yeyote ya kusafiri au kuhama
  - Kwanini? Na kipi kinapelekea kuhama?
- Matumaini au mipango kwa ajili ya mtoto – Kipi unashauku nacho? Kipi unakihofia?

1. **Hitimisho**

Una mawazo gani kuhusiana na jinsi ambavyo tunaweza kuwasaidia wanawake wenye maambukizi ya VVU kama wewe kubaki kwenye huduma baada ya kujifungua?

Umejisikiaje kuongea na mimi sasa baada ya kupata mtoto?

- Kipi ambacho kimekuwa cha msaada kwenye mazungumszo yetu
- Unafikiri mazungumzo haya yanaweza kuwasaidia wanawake wengine

Je una jambo lolote ungependa kuongezea kabla hatujahitimisha mazungumzo yetu?

Asante kwa kutumia muda wako kuzungumza na mimi. Mtizamo na uzoefu wako utatusaidia kutoa msaada kwa wamama waliojifungua katika jamii yetu.
